# After-effects of thixotropic conditionings on operational chest wall and compartmental volumes of patients with Parkinson’s disease

**DOI:** 10.1371/journal.pone.0275584

**Published:** 2022-10-14

**Authors:** Maria Clara Rodrigues de Góes, Antonio Sarmento, Illia Lima, Marina Lyra, Cristiane Lima, Andrea Aliverti, Vanessa Resqueti, Guilherme A. F. Fregonezi

**Affiliations:** 1 PneumoCardioVascular Laboratory—Hospital Universitário Onofre Lopes, Empresa Brasileira de Serviços Hospitalares (EBSERH) & Laboratório de Inovação Tecnológica em Reabilitação, Departamento de Fisioterapia, Universidade Federal do Rio Grande do Norte (UFRN), Natal, Brazil; 2 Faculdade de Ciências da Saúde do Trairí (FACISA), Universidade Federal do Rio Grande do Norte (UFRN), Santa Cruz, Brazil; 3 Dipartimento di Elettronica, Informazione e Bioingegneria, Politecnico di Milano, Milan, Italy; University of Western Ontario, CANADA

## Abstract

Individuals with Parkinson’s disease (PD) present respiratory dysfunctions, mainly due to decreased chest wall expansion, which worsens with the course of the disease. These findings contribute to the restrictive respiratory pattern and the reduction in chest wall volume. According to literature, inspiratory muscle thixotropic conditioning maneuvers may improve lung volumes in these patients. The study aimed to determine the after-effects of respiratory muscle thixotropic maneuvers on breathing patterns and chest wall volumes of PD. A crossover study was performed with twelve patients with PD (8 males; mean age 63.9±8.8 years, FVC_%pred_ 89.7±13.9, FEV_1%pred_ 91.2±15, FEV_1_/FVC_%pred_ 83.7±5.7). Chest wall volumes were assessed using OEP during thixotropic maneuvers. Increases in EIV_CW_ (mean of 126mL, p = 0.01) and EEV_CW_ (mean of 150mL, p = 0.005) were observed after DI_TLC_ (deep inspiration from total lung capacity) due to increases in pulmonary (RCp) and abdominal (RCa) ribcage compartments. Changes in ICo_TLC_ (inspiratory contraction from TLC) led to significant EIV_CW_ (mean of 224mL, p = 0.001) and EEV_CW_ (mean of 229mL, p = 0.02) increases that were mainly observed in the RCp. No significant changes were found when performing DE_RV_ (deep expiration from residual volume) and ICo_RV_ (Inspiratory contraction from RV). Positive correlations were also observed between the degree of inspiratory contraction during ICo_TLC_ and EEV_RCp_ (*rho* = 0.613, p = 0.03) and EIV_RCp_ (*rho* = 0.697, p = 0.01) changes. Thixotropy conditioning of inspiratory muscles at an inflated chest wall volume increases EIV_CW_ and EEV_CW_ in the ten subsequent breaths in PD patients. These maneuvers are easy to perform, free of equipment, low-cost, and may help patients improve chest wall volumes during rehabilitation.

## Introduction

Parkinson’s disease (PD) is a common and complex neurological disorder. The main clinical feature of PD is related to postural and movement disorders due to the destruction of the substantia nigra and loss of dopaminergic neurons [1, 2]. Nevertheless, respiratory dysfunction (e.g., impaired upper airway function and decreased chest wall compliance) leads to problems that predispose to pneumonia, the leading cause of mortality in PD [1, 2]. The restrictive pattern is the main spirometric finding [1, 3], and respiratory muscle strength may also decrease with the course of the disease [4]. Some studies demonstrate that respiratory muscles can be impaired even in the early stages of the disease [5]. Together, these events contribute to the restrictive pattern and reduced chest wall compliance and volume [6–9].

In restrictive diseases, reduced chest wall compliance is associated with decreased chest wall volumes. Therefore, new techniques were proposed in the medical literature to improve chest wall volumes based on the physiological behavior of muscles, such as thixotropy. Thixotropy is a biophysical property that makes the stiffness and resting tension at a given muscle length dependent on previous movements and contractions. The history-dependent passive properties of inspiratory muscles are one relevant component of expiration since expiratory movements need to stretch inspiratory muscles to reduce chest wall volume [10].

Homma and Hagbarth [10] proposed that the thixotropy conditioning of respiratory muscles could temporarily increase end-expiratory volume during resting breathing. This hypothesis is based on the muscle thixotropy characteristic: mechanical properties of the muscle change with movement and recover after the movement stops. In this sense, inspiratory muscles must be brought from an intermediate to a shorter length, and an inspiratory muscle contraction is performed to detach cross-bridges. After the effort, the muscle is held at the acquired length for a few seconds and brought back slowly to its intermediate length [11–13]. As a result, passive stiffness and resting tension in inspiratory muscles are combined with a slackness of expiratory muscles [14]. In a recent study published by Lima et al., it was possible to observe a significant increase in end-inspiratory volume (EIV) after the contraction of inspiratory muscles from residual volume (RV) and total lung capacity (TLC) in healthy individuals [15].

In this context, the present study aimed to determine whether thixotropic conditionings of inspiratory muscles would acutely increase the operational chest wall volumes of PD patients. For this, we used optoelectronic plethysmography (OEP), a system that accurately measures chest wall volumes in a non-invasive way and on a three-compartmental basis (i.e., pulmonary ribcage [RCp], abdominal ribcage [RCa], and abdomen [AB]) [16]. Additionally, the volumes acquired by this system during inspiration and expiration to produce an index of overall inspiratory and expiratory muscle length [17, 18], respectively, which facilitates the observation of the thixotropic after-effects.

## Material and methods

### Subjects

This is a randomized crossover study conducted following the Declaration of Helsinki and approved by the ethics committee of the Hospital Universitário Onofre Lopes (HUOL/EBSERH—Brazil) under number 1.662.429/2016. Patients were recruited between July 2015 and December 2016. Before participation in the study, all subjects gave written, signed, and informed consent.

Stable patients with a confirmed diagnosis of PD by clinical examination (experienced neurologist), non- and ex-smokers, and with a maximal score of II on the Hoehn & Yahr scale [19] were recruited from the neurology outpatient clinics of the hospital mentioned above. Patients who self-reported cardiopulmonary or musculoskeletal diseases were excluded. The study sample was calculated based on a pilot study considering the variables of interest. All patients during the study maintained their pharmacological treatment defined by the neurologist, and assessments were always conducted at the same time of day. Ex-smoker patients were omitted. Those patients who missed the assessments or failed to perform tests were excluded from the study.

### Spirometry

A Koko Digidoser spirometer was used to assess pulmonary function according to ATS/ERS recommendations [20]. Forced vital capacity (FVC), forced expiratory volume in the first second (FEV_1_), and FEV_1_/FVC ratio was considered in their absolute and percentage of predicted values for the Brazilian population [21]; the best curve obtained from three acceptable maneuvers was considered in the study. As reduced FVC does not confirm a restrictive pulmonary defect, this condition was inferred if Forced Vital Capacity was reduced, FEV_1_/FVC increased (between 85–90% of predicted values), and the flow-volume curve presented a convex pattern [22].

### Respiratory muscle strength

Respiratory muscle strength was assessed through MIP and MEP using a digital manovacuometer according to ATS/ERS recommendations [23]. Inspiratory muscle strength was also assessed through sniff nasal inspiratory pressure test. All values obtained were compared with reference values [24, 25]. MEP/MIP ratio was also reported in their absolute and percentage of predicted values [26]. The lower limits of normal values were used to determine respiratory muscle weakness [27].

### Chest wall and compartmental volumes

Chest wall (V_CW_) and compartmental volumes (V_RCp_, V_RCa_, and V_AB_) were assessed using OEP. This system consisted of six TV cameras (three in front and three behind the subject), previous calibrated at a frequency of 60 frames∙s^-1^that captured the signal of 89 photosensitive markers placed at predetermined points of the trunk surface [28]. After data collection, volumes were obtained through a mathematical model based on the Gaussian Theorem [16].

From OEP, chest wall, compartmental volumes, operational end-inspiratory (EIV_CW_, EIV_RCp_, EIV_RCa_, and EIV_AB_) and end-expiratory volumes (EEV_CW_, EEV_RCp_, EEV_RCa_, and EEV_AB_), and variables of breathing pattern (inspiratory and expiratory times) were analyzed. Shortening velocity index of the diaphragm (Vt,ab/inspiratory time), inspiratory ribcage (Vt, rcp/inspiratory time), and expiratory muscles (Vt, rcp/expiratory time) were also calculated according to Aliverti et al. [29].

### Pneumotachography

A heated pneumotachograph connected to a mouthpiece measured pressure during the thixotropic conditionings. The pressure transducer was calibrated before tests using a water manometer and measuring positive and negative pressure variations from 0 to 100 cmH_2_O, with intervals of 5 seconds in between. During data acquisition, patients used a nose clip and were instructed to perform the thixotropic conditionings while wearing a mouthpiece. Pressure data was always acquired synchronously with the OEP data.

### Thixotropic conditionings

One pneumatic valve was coupled with the pneumotachograph, and an automated controller was used to manually trigger the opening and occlusion of the valves during the conditioning maneuvers. Mouth pressure (in cmH_2_O) was acquired during all thixotropic conditionings to establish the intensity level of the maneuvers during inspiratory contraction. These values were described as a percentage of predicted MIP.

Before each conditioning, patients were taught how to perform the thixotropic maneuvers. Afterward, patients were allowed to rest for 10 minutes before starting the experiment. All thixotropic conditionings performed by the patients initiated from an inflated (TLC) or deflated (RV) chest wall [10]. For this study, four different thixotropic maneuvers were performed: deep inspiration until Total Lung Capacity (DI_TLC_); deep expiration until Residual Volume (DE_RV_); inspiratory contraction from Total Lung Capacity (ICo_TLC_); and inspiratory contraction from Residual Volume (ICo_RV_). For DI_TLC_ and DE_RV_, patients were instructed to fully inflate or deflate, respectively, the chest wall and then the shutter of the pneumatic valve closed the airway for five seconds. At the same time, the patient remained in the same chest wall position without breathing. Right after, voluntary muscle relaxation was performed at the acquired chest wall position for three seconds, and then the shutter was reopened, allowing the subject to resume breathing. For ICo_TLC_ and ICo_RV_, the pneumatic valve closed the airway after chest wall inflation or deflation. The patients were asked to make a forceful inspiratory contraction using the ribcage muscles for 5 seconds. After the effort, voluntary muscle relaxation was performed, the airway was opened, and breathing was resumed [10, 14].

### Study design and data analysis

**Fig 1:**
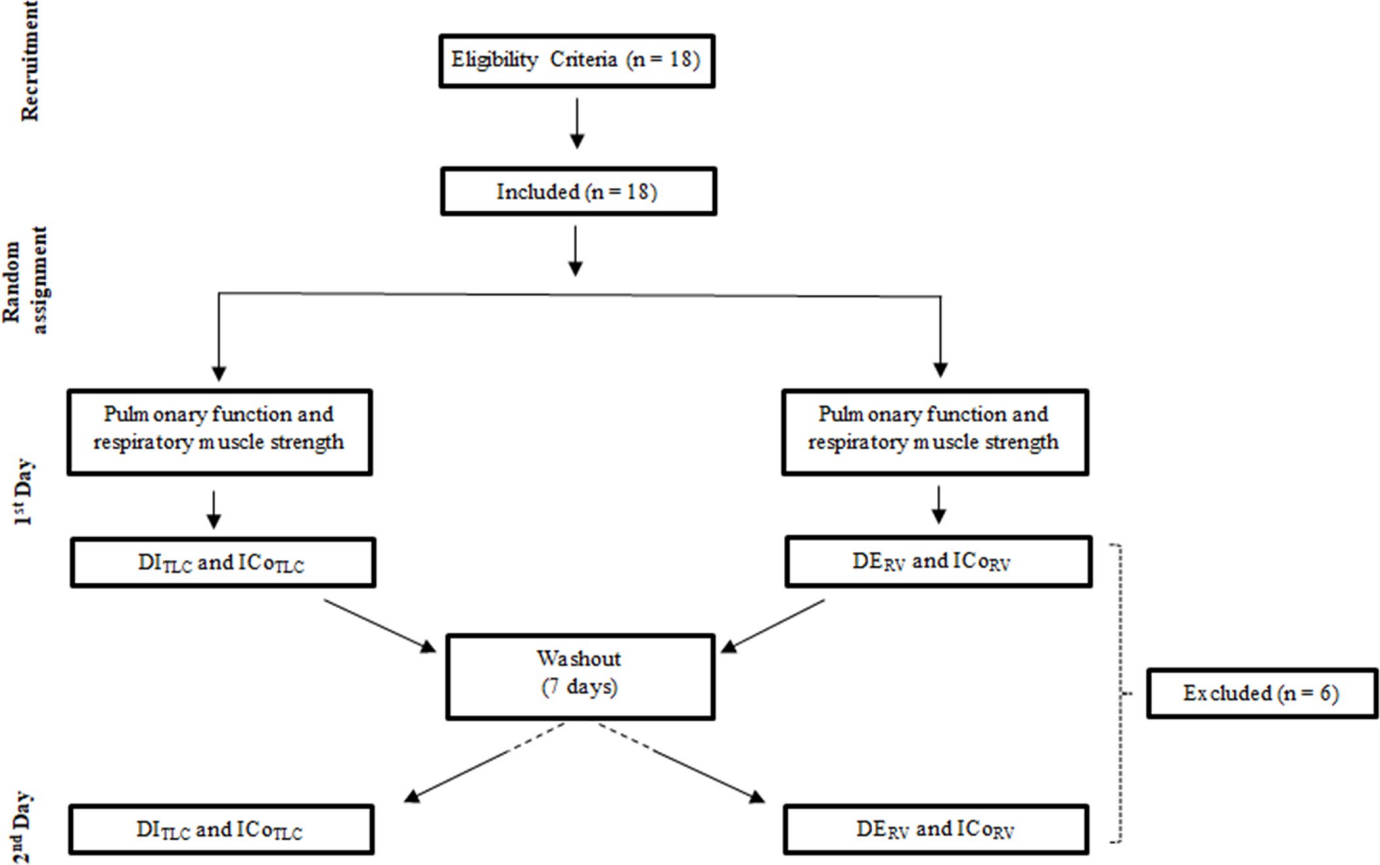
Flowchart of the study. DI_TLC_: Deep inspiration from total lung capacity; ICo_TLC_: Inspiratory contraction from full lung capacity; DE_RV_: Deep inspiration from residual volume; ICo_RV_: Inspiratory contraction from residual volume.

Measurements were performed in two different visits with a one-week interval in between. In the first visit, anthropometric, lung function, and respiratory muscle strength data were acquired, and two thixotropic conditionings with 30 minutes in between were performed. In the second visit, the remaining two thixotropic conditionings were performed with the same time interval between them. Thixotropic conditionings were randomized using the website "randomization.com" (Fig 1). In each subject, the after-effects of the maneuvers on operational volumes were studied in detail immediately after each contraction (the first sten respiratory cycles) and compared to baseline.

After positioning the pneumotachograph and OEP markers, patients were asked to wear a nose clip and sit on a chair with the arms supported away from the sides of the body to allow the visualization of the markers by the TV cameras. After data acquisition during one minute of quiet breathing (QB), patients were asked to perform one of the above-mentioned thixotropic maneuvers. Immediately after each maneuver and consequently reopening the shuttle valve, patients reassumed breathing, and one minute of QB was recorded.

For data analysis, the chest wall and compartmental EIV and EEV, shortening velocity indexes, inspiratory and expiratory times, and total time of respiratory cycle were analyzed on a breath-by-breath basis during the first ten respiratory cycles immediately after each maneuver and compared with baseline values obtained during one minute of QB performed before each conditioning maneuver.

### Statistical analyses

The sample size was calculated a priori according to EEV_CW_ changes after ICo_TLC_, collected from 4 volunteers. Effect sizes were calculated (ɳ_p_ = .417 and Cohen’s f = .845) considering a significance level of < .05 and a statistical power of .90, and the optimal number was estimated as 12 patients.

Results are expressed as mean ± SD otherwise stated. Data normality or symmetry was tested using the Shapiro-Wilk test. Unpaired t-test or Mann–Whitney test was used to compare the after-effects of EIV and EEV between thixotropic conditionings performed from the same chest wall position. One-way ANOVA for repeated measures or Friedman test was used to compare OEP data obtained in the ten respiratory cycles with those during QB. In the case of statistical significance, Bonferroni’s or Dunn’s post hoc tests were applied to identify differences. Relationships between mouth pressure during conditionings and the mean chest wall and operational volume changes after the maneuvers were studied using Spearman’s *rho* correlation coefficient.

Descriptive and inferential analyses were conducted using GraphPad Prism^®^ Software, version 7.04 (Graphpad Inc., La Jolla, United States). P-values < .05 were considered statistically significant.

## Results

### Participants

Eighteen PD patients were recruited. Six were excluded due to their inability to understand or perform the thixotropic conditionings (Fig 1). The final sample comprised twelve PD patients (8 males and 4 females) characterized by restrictive patterns. Mean MIP_%pred_ (83.5±33.3) and SNIP_%pred_ (63.6±15.6) were lower than the mean MEP_%pred_ (100.6±22.2) (Table 1); mean MIP/MEP_%pred_ ratio was 1.35±0.6.

**Table 1:** Anthropometric and spirometric data.

Patients	Gender	Age _years_	BMI _kg/m_^2^	FVC _L_	FVC _%pred_	FEV_1 L_	FEV_1%pred_	FEV_1_/FVC _%pred_	MIP _cmH2O_	MIP _%pred_	MEP _cmH2O_	MEP _%pred_	SNIP _cmH2O_	SNIP _%pred_
#1	M	66	21.3	3.76	82	3.32	94	114.6	96	93.6	78	68.7	61	58.3
#2	M	76	24.9	3.9	96.2	2.91	95.2	98.9	116	122.7	116	111.8	57	57.1
#3	M	67	30.5	3.48	88.6	2.81	91.6	103.3	41	40.3	88	79.2	71	68.2
#4	M	72	32.3	3.23	89.2	2.74	97.6	109.44	81	82.91	116	108.4	66	65
#5	M	67	20	4.1	94.4	3.16	94	99.5	130	117.1	102	100.3	105	98
#6	M	63	32.7	3.9	102.6	3.15	104	101.3	69	65.8	140	122.5	80	75.5
#7	M	69	26.6	3.06	83.9	2.15	74.9	89.2	154	153.8	116	106	55	53.3
#8	M	68	19.6	3.96	85.2	3.01	84.7	99.4	67	66.4	103	93.4	51	49.21
#9	F	62	18.3	2.51	87.7	1.97	86.1	95.4	44	55	63	81	40	45.5
#10	F	42	23.1	2.19	60.2	1.86	62.5	103.7	57	63.4	75	83.3	66	69.5
#11	F	59	29.3	3.76	120	3.1	123.8	103.3	70	85.9	81	101.7	66	74.2
#12	F	56	23.5	2.6	86.2	2.1	86	99.9	46	55.4	123	151	42	46.7
Mean		63.9	25.2	3.37	89.7	2.69	91.2	101.5	80.92	83.5	100.1	100.6	63.33	63.6
SD		8.8	5.1	0.64	13.9	0.52	15	6.4	36.20	33.3	23.15	22.2	17.48	15.6

In the left column are the participant’s numbers. FVC: Forced vital capacity; FEV_1_: Forced expiratory volume in the first second; MIP: Maximum inspiratory pressure; MEP: Maximum expiratory pressure; SNIP: Sniff nasal inspiratory pressure; SD: Standard deviation; m: Meters; kg: Kilograms; L: Liters; cmH_2_O: centimeters of water; %pred: Percentage of predicted; F: Female; M: Male. Shapiro-Wilk test was used to determine normality or symmetry.

### Chest wall and compartmental volumes

#### Thixotropic conditionings from TLC

**Fig 2:**
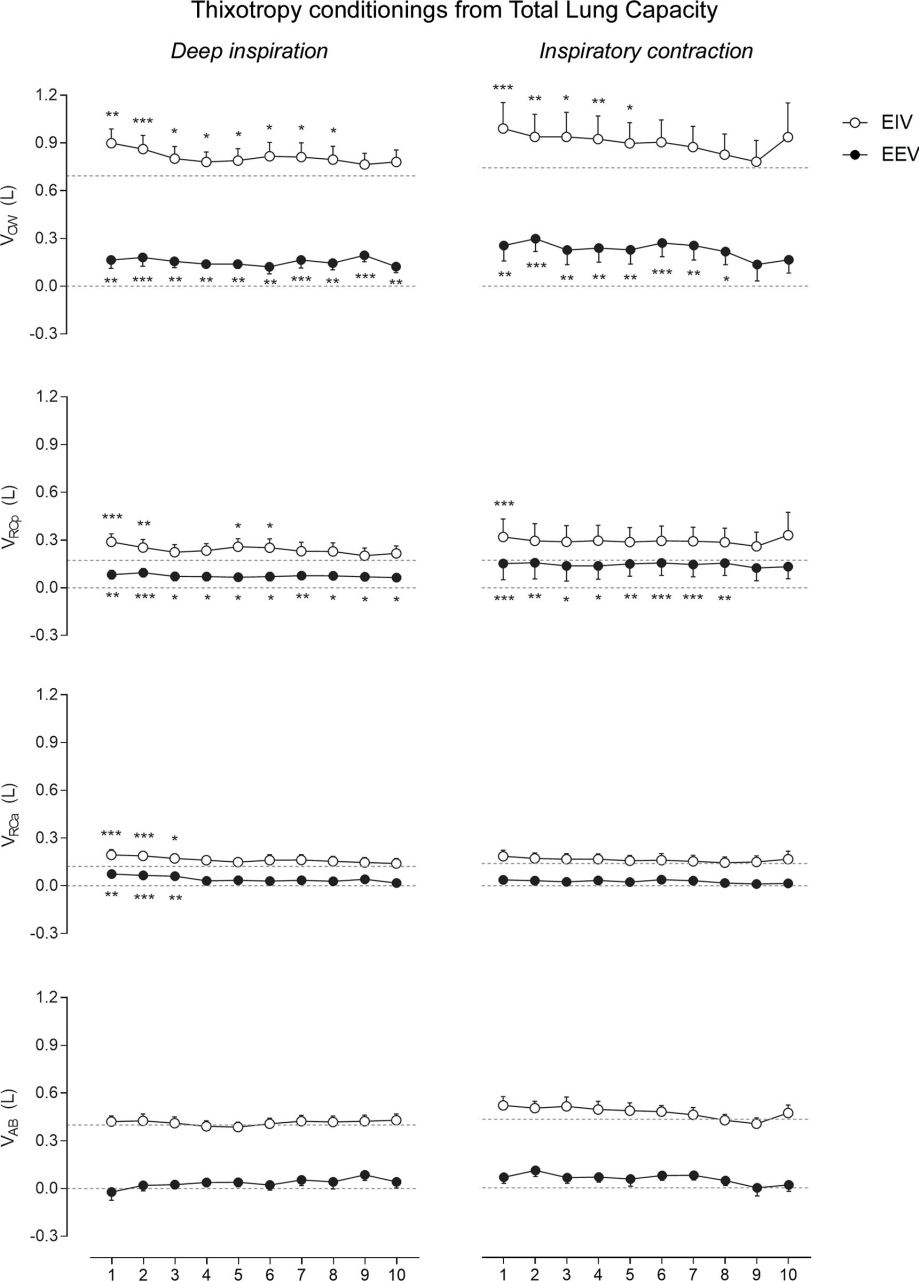
After-effects of thixotropic conditionings performed from total lung capacity on end-inspiratory (EIV) and end-expiratory (EEV) volumes of the chest wall (CW) and its compartments (pulmonary ribcage [RCp], abdominal ribcage [RCa] and abdominal [AB]). Data of the first ten respiratory cycles (x-axis) immediately after the conditionings were compared with mean values (mean of the 60 seconds) of quiet breathing (dotted lines) performed before the maneuvers. Data are shown as mean ± SE. L: Liters; ***p < .0001, **p < .001, and *p < .05 compared with quiet breathing using one-way ANOVA (CW, RCp, and RCa) and Friedman test (AB), and Dunn’s post hoc test.

As shown in Fig 2, both DI_TLC_ and ICo_TLC_ conditionings significantly increased EEV_CW_ and EIV_CW_ in PD patients. Increases in EEV_CW_ after DI_TLC_ (mean of 150 mL, p = .005) were mainly due to increases in EEV_RCp_ (p = .03) and EEV_RCa_ (p = .02); whereas increases after ICo_TLC_ (mean of 229 mL, p = .02) were due to changes in the RCp compartment only (p = .03). Regarding EIV_CW_, changes after DI_TLC_ (mean of 126 mL, p = .01) and ICo_TLC_ (mean of 224 mL, p = .001) conditionings were mainly due to increases in EIV_RCp_; however, the duration was shorter than the after-effects observed in EEV.

None of the thixotropic conditionings performed from TLC significantly changed the operational volumes of the AB compartment. Additionally, no significant changes were observed in V_CW_, V_RCp_, V_RCa_, and V_AB_ (Table 2). No significant differences were observed when comparing the volume changes of both DI_TLC_ and ICo_TLC_ conditionings.

**Table 2:** Effects of deep inspiration (A) and inspiratory contraction (B) from total lung capacity on chest wall volumes.

**A)**											
	**Quiet breathing**	**1° cycle**	**2° cycle**	**3° cycle**	**4° cycle**	**5° cycle**	**6° cycle**	**7° cycle**	**8° cycle**	**9° cycle**	**10° cycle**
V_CW (L)_	0.693 ± 0.22	0.763 ± 0.35	0.682 ± 0.34	0.645 ± 0.25	0.641 ± 0.22	0.649 ± 0.19	0.695 ± 0.24	0.646 ± 0.20	0.650 ± 0.21	0.570 ± 0.16	0.658 ± 0.22
V_RCp (L)_	0.172 ± 0.15	0.205 ±0.16	0.158 ± 0.14	0.152 ± 0.12	0.163 ± 0.13	0.191 ± 0.14	0.181 ± 0.16	0.154 ± 0.16	0.152 ± 0.15	0.132 ± 0.11	0.151 ± 0.14
V_RCa (L)_	0.122 ± 0.10	0.120 ± 0.06	0.122 ± 0.06	0.112 ± 0.06	0.129 ± 0.08	0.115 ± 0.06	0.132 ± 0.10	0.127 ± 0.10	0.125 ± 0.09	0.105 ± 0.06	0.122 ± 0.09
V_AB (L)_	0.399 ± 0.15	0.438 ± 0.18	0.401 ± 0.21	0.381 ± 0.18	0.348 ± 0.16	0.342 ± 0.12	0.381 ± 0.13	0.364 ± 0.11	0.371 ± 0.13	0.332 ± 0.12	0.384 ± 0.14
Ti _(s)_	1.55 ± 0.25	1.63 ± 0.39	1.72 ± 0.78	1.61 ± 0.71	1.46 ± 0.29	1.44 ± 0.27	1.50 ± 0.30	1.47 ± 0.33	1.46 ± 0.25	1.30 ± 0.24	1.50 ± 0.30
Te _(s)_	2.01 ± 0.62	2.21 ± 1.28	2.39 ± 1.23	2.12 ± 1.02	2.03 ± 0.70	1.97 ± 0.62	1.99 ± 0.50	2.05 ± 0.78	2.02 ± 0.50	1.99 ± 0.52	2.01 ± 0.74
Ttot _(s)_	3.57 ± 0.80	3.84 ± 1.52	4.11 ± 1.82	3.74 ± 1.53	3.49 ± 0.89	3.41 ± 0.80	3.49 ± 0.59	3.52 ± 1.07	3.48 ± 0.61	3.30 ± 0.72	3.51 ± 0.86
ΔV_RCp_/Ti _(L/s)_	0.11 ± 0.10	0.13 ± 0.11	0.10 ± 0.09	0.09 ± 0.08	0.11 ± 0.10	0.13 ± 0.09	0.11 ± 0.09	0.10 ± 0.10	0.10 ± 0.09	0.10 ± 0.09	0.09 ± 0.09
ΔV_AB_/Ti _(L/s)_	0.25 ± 0.08	0.27 ± 0.11	0.23 ± 0.09	0.24 ± 0.10	0.23 ± 0.08	0.24 ± 0.07	0.25 ± 0.06	0.25 ± 0.08	0.25 ± 0.08	0.26 ± 0.10	0.25 ± 0.08
ΔV_AB_/Te _(L/s)_	0.22 ± 0.12	0.24 ± 0.15	0.19 ± 0.15	0.21 ± 0.13	0.19 ± 0.13	0.19 ± 0.10	0.21 ±0.11	0.20 ± 0.10	0.20 ± 0.11	0.17 ± 0.07	0.21 ±0.10
**B)**											
	**Quiet breathing**	**1° cycle**	**2° cycle**	**3° cycle**	**4° cycle**	**5° cycle**	**6° cycle**	**7° cycle**	**8° cycle**	**9° cycle**	**10° cycle**
V_CW (L)_	0.744 ± 0.31	0.766 ± 0.35	0.671 ± 0.33	0.741 ± 0.33	0.715 ± 0.34	0.700 ± 0.31	0.663 ± 0.26	0.649 ± 0.32	0.639 ± 0.25	0.676 ± 0.25	0.799 ± 0.52
V_RCp (L)_	0.173 ± 0.15	0.166 ± 0.17	0.137 ± 0.16	0.151 ± 0.12	0.158 ± 0.14	0.136 ± 0.13	0.138 ± 0.12	0.146 ± 0.16	0.131 ± 0.11	0.136 ± 0.11	0.200 ± 0.26
V_RCa (L)_	0.140 ± 0.11	0.147 ± 0.14	0.140 ± 0.14	0.141 ± 0.11	0.132 ± 0.10	0.134 ± 0.11	0.122 ± 0.10	0.121 ± 0.10	0.128 ± 0.09	0.137 ± 0.10	0.150 ± 0.16
V_AB (L)_	0.431 ± 0.13	0.451 ± 0.14	0.392 ± 0.13	0.447 ± 0.21	0.424 ± 0.20	0.429 ± 0.17	0.402 ± 0.14	0.381 ± 0.15	0.379 ± 0.13	0.402 ± 0.19	0.450 ± 0.22
Ti _(s)_	1.49 ± 0.40	1.67 ± 0.61	1.39 ± 0.32	1.45 ± 0.42	1.39 ± 0.36	1.43 ± 0.49	1.69 ± 1.03	1.27 ± 0.35	1.32 ± 0.31	1.27 ± 0.24	1.46 ± 0.50
Te _(s)_	2.06 ± 0.55	2.03 ± 0.86	2.28 ± 0.70	2.08 ± 1.10	2.21 ± 1.12	1.92 ± 0.65	1.97 ± 0.65	2.18 ± 1.03	1.95 ± 0.73	1.90 ± 0.47	1.80 ± 0.55
Ttot _(s)_	3.55 ± 0.93	3.70 ± 1.39	3.67 ± 1.00	3.53 ± 1.48	3.61 ± 1.41	3.36 ± 1.00	3.66 ± 1.15	3.46 ± 1.27	3.27 ± 1.00	3.17 ± 0.67	3.26 ± 0.95
ΔV_RCp_/Ti _(L/s)_	0.12 ± 0.11	0.10 ± 0.09	0.10 ± 0.10	0.10 ± 0.08	0.10 ± 0.08	0.09 ± 0.09	0.09 ± 0.09	0.10 ± 0.10	0.10 ± 0.09	0.11 ± 0.10	0.13 ± 0.14
ΔV_AB_/Ti _(L/s)_	0.28 ± 0.07	0.28 ± 0.09	0.29 ± 0.12	0.30 ± 0.11	0.29 ± 0.08	0.29 ± 0.07	0.28 ± 0.11	0.29 ± 0.05	0.28 ± 0.06	0.30 ± 0.09	0.30 ± 0.08
ΔV_AB_/Te _(L/s)_	0.21 ± 0.06	0.25 ± 0.12	0.18 ± 0.07	0.23 ± 0.09	0.20 ± 0.07	0.22 ± 0.05	0.21 ± 0.06	0.19 ± 0.06	0.20 ± 0.04	0.20 ± 0.06	0.25 ± 0.09

Data are shown as mean ± SD. S: Seconds; L: Liters.

#### Thixotropic conditionings from RV

**Fig 3:**
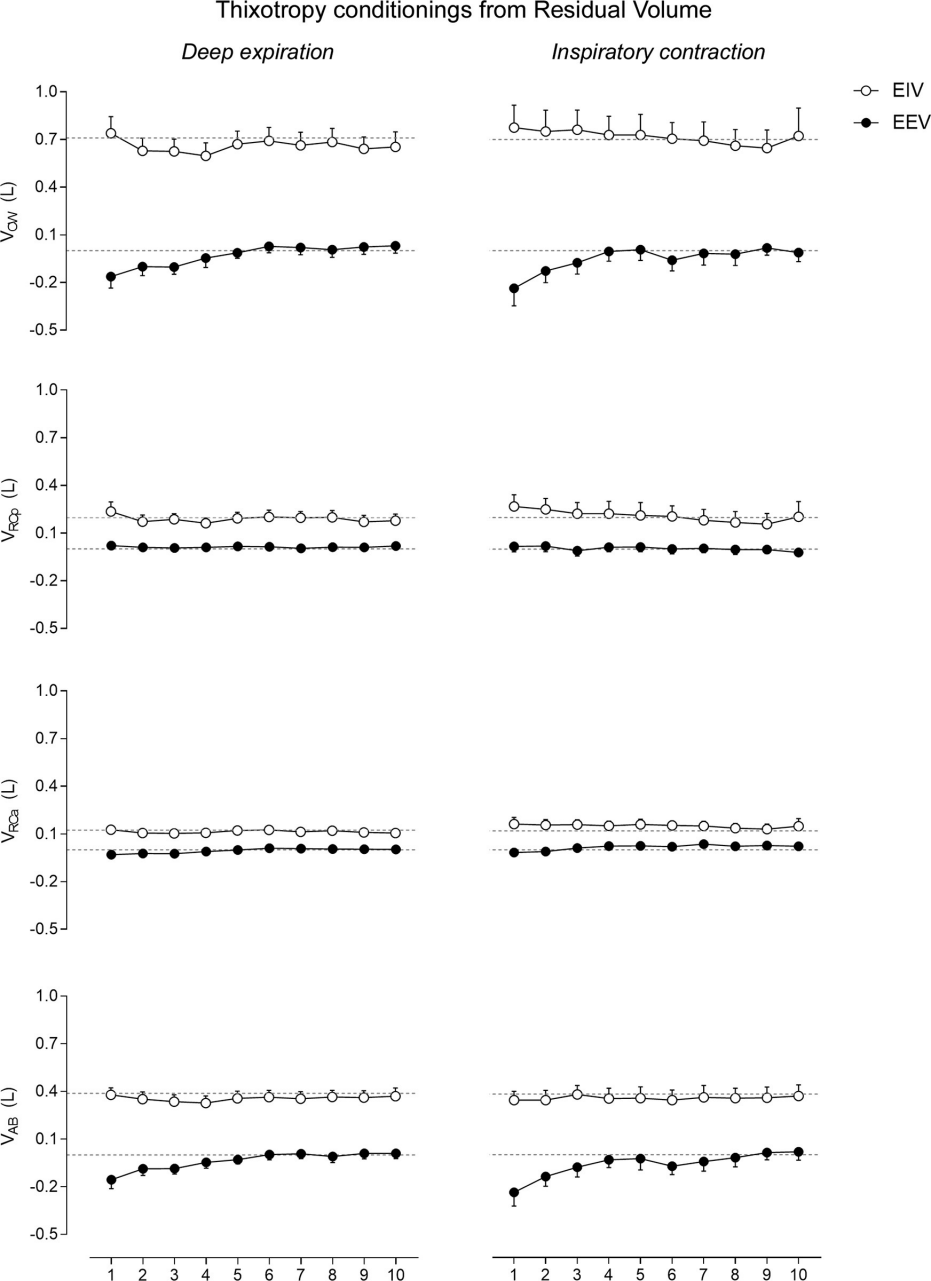
After-effects of thixotropic conditionings performed from residual volume on end-inspiratory (EIV) and end-expiratory (EEV) volumes of the chest wall (CW) and its compartments (pulmonary ribcage [RCp], abdominal ribcage [RCa] and abdominal [AB]). Data of the first ten respiratory cycles (x-axis) immediately after the conditionings were compared with mean values (mean of 60 seconds) of quiet breathing (dotted lines) performed before the maneuvers. Data are shown as mean ± SE. L: Liters.

No significant differences were observed in chest wall and operational volumes after DI_RV_ or ICo_RV_ conditionings (Fig 3 and Table 3). No significant differences were observed between the after-effects of DI_RV_ and ICo_RV_ conditionings.

**Table 3:** Effects of deep expiration (A) and inspiratory contraction (B) from the residual volume on the chest wall volumes.

**A)**											
	**Quiet breathing**	**1° cycle**	**2° cycle**	**3° cycle**	**4° cycle**	**5° cycle**	**6° cycle**	**7° cycle**	**8° cycle**	**9° cycle**	**10° cycle**
V_CW (L)_	0.693 ± 0.22	0.763 ± 0.35	0.682 ± 0.34	0.645 ± 0.25	0.641 ± 0.22	0.649 ± 0.19	0.695 ± 0.24	0.646 ± 0.20	0.650 ± 0.21	0.570 ± 0.16	0.658 ± 0.22
V_RCp (L)_	0.172 ± 0.15	0.205 ±0.16	0.158 ± 0.14	0.152 ± 0.12	0.163 ± 0.13	0.191 ± 0.14	0.181 ± 0.16	0.154 ± 0.16	0.152 ± 0.15	0.132 ± 0.11	0.151 ± 0.14
V_RCa (L)_	0.122 ± 0.10	0.120 ± 0.06	0.122 ± 0.06	0.112 ± 0.06	0.129 ± 0.08	0.115 ± 0.06	0.132 ± 0.10	0.127 ± 0.10	0.125 ± 0.09	0.105 ± 0.06	0.122 ± 0.09
V_AB (L)_	0.399 ± 0.15	0.438 ± 0.18	0.401 ± 0.21	0.381 ± 0.18	0.348 ± 0.16	0.342 ± 0.12	0.381 ± 0.13	0.364 ± 0.11	0.371 ± 0.13	0.332 ± 0.12	0.384 ± 0.14
Ti _(s)_	1.55 ± 0.25	1.63 ± 0.39	1.72 ± 0.78	1.61 ± 0.71	1.46 ± 0.29	1.44 ± 0.27	1.50 ± 0.30	1.47 ± 0.33	1.46 ± 0.25	1.30 ± 0.24	1.50 ± 0.30
Te _(s)_	2.01 ± 0.62	2.21 ± 1.28	2.39 ± 1.23	2.12 ± 1.02	2.03 ± 0.70	1.97 ± 0.62	1.99 ± 0.50	2.05 ± 0.78	2.02 ± 0.50	1.99 ± 0.52	2.01 ± 0.74
Ttot _(s)_	3.57 ± 0.80	3.84 ± 1.52	4.11 ± 1.82	3.74 ± 1.53	3.49 ± 0.89	3.41 ± 0.80	3.49 ± 0.59	3.52 ± 1.07	3.48 ± 0.61	3.30 ± 0.72	3.51 ± 0.86
ΔV_RCp_/Ti _(L/s)_	0.11 ± 0.10	0.13 ± 0.11	0.10 ± 0.09	0.09 ± 0.08	0.11 ± 0.10	0.13 ± 0.09	0.11 ± 0.09	0.10 ± 0.10	0.10 ± 0.09	0.10 ± 0.09	0.09 ± 0.09
ΔV_AB_/Ti _(L/s)_	0.25 ± 0.08	0.27 ± 0.11	0.23 ± 0.09	0.24 ± 0.10	0.23 ± 0.08	0.24 ± 0.07	0.25 ± 0.06	0.25 ± 0.08	0.25 ± 0.08	0.26 ± 0.10	0.25 ± 0.08
ΔV_AB_/Te _(L/s)_	0.22 ± 0.12	0.24 ± 0.15	0.19 ± 0.15	0.21 ± 0.13	0.19 ± 0.13	0.19 ± 0.10	0.21 ±0.11	0.20 ± 0.10	0.20 ± 0.11	0.17 ± 0.07	0.21 ±0.10
**B)**											
	**Quiet breathing**	**1° cycle**	**2° cycle**	**3° cycle**	**4° cycle**	**5° cycle**	**6° cycle**	**7° cycle**	**8° cycle**	**9° cycle**	**10° cycle**
V_CW (L)_	0.700 ± 0.32	1.011 ± 0.48	0.877 ± 0.41	0.837 ± 0.39	0.732 ± 0.30	0.722 ± 0.46	0.763 ± 0.35	0.710 ± 0.41	0.683 ± 0.39	0.628 ± 0.36	0.735 ± 0.60
V_RCp (L)_	0.198 ± 0.18	0.251 ± 0.21	0.230 ± 0.20	0.232 ± 0.20	0.219 ± 0.19	0.206 ± 0.24	0.213 ± 0.19	0.192 ± 0.22	0.194 ± 0.21	0.180 ± 0.19	0.256 ± 0.29
V_RCa (L)_	0.118 ± 0.08	0.178 ± 0.14	0.166 ± 0.14	0.147 ± 0.12	0.126 ± 0.11	0.134 ± 0.13	0.134 ± 0.11	0.114 ± 0.10	0.114 ± 0.10	0.102 ± 0.10	0.126 ± 0.17
V_AB (L)_	0.382 ± 0.14	0.580 ± 0.32	0.480 ± 0.23	0.457 ± 0.20	0.386 ± 0.17	0.380 ± 0.19	0.416 ± 0.17	0.403 ± 0.21	0.374 ± 0.19	0.344 ± 0.16	0.351 ± 0.18
Ti _(s)_	1.42 ± 0.27	1.33 ± 0.38	1.43 ± 0.31	1.49 ± 0.41	1.34 ± 0.34	1.21 ± 0.31	1.49 ± 0.29	1.37 ± 0.33	1.34 ± 0.36	1.20 ± 0.32	1.35 ± 0.46
Te _(s)_	2.13 ± 0.67	1.77 ± 0.50	2.10 ± 1.10	2.02 ± 0.83	2.28 ± 0.90	2.43 ± 1.70	1.95 ± 0.58	1.92 ± 0.76	1.99 ± 0.64	1.81 ± 0.68	1.78 ± 0.74
Ttot _(s)_	3.56 ± 0.86	3.11 ± 0.84	3.53 ± 1.35	3.51 ± 1.13	3.63 ± 1.20	3.65 ± 1.92	3.45 ± 0.72	3.30 ± 1.01	3.34 ± 0.82	3.02 ± 0.98	3.14 ± 1.05
ΔV_RCp_/Ti _(L/s)_	0.14 ± 0.12	0.18 ± 0.14	0.16 ± 0.14	0.16 ± 0.13	0.17 ± 0.16	0.16 ± 0.15	0.14 ± 0.13	0.13 ± 0.14	0.14 ± 0.16	0.14 ± 0.13	0.16 ± 0.15
ΔV_AB_/Ti _(L/s)_	0.26 ± 0.07	0.41 ± 0.17	0.35 ± 0.18	0.31 ± 0.13	0.28 ± 0.11	0.30 ± 0.11	0.28 ± 0.10	0.27 ± 0.09	0.26 ± 0.08	0.28 ± 0.09	0.24 ± 0.07
ΔV_AB_/Te _(L/s)_	0.18 ± 0.05	0.31 ± 0.13	0.24 ± 0.10	0.24 ± 0.09	0.18 ± 0.09	0.19 ± 0.11	0.22 ± 0.10	0.21 ± 0.10	0.20 ± 0.11	0.19 ± 0.07	0.20 ± 0.08

Data are shown as mean ± SD. Seconds; L: Liters

#### Breathing pattern and shortening velocity indexes

Compared with QB, no significant differences in inspiratory and expiratory times, total time of the respiratory cycle, and shortening velocity indexes were found after the conditionings (Tables 2 and 3).

#### Correlations

Mean mouth pressure during ICoTLC and ICoRV were -28.8 ±13.2 and -57.9 ±15.1 cmH_2_O, respectively. No significant relationships were observed between chest wall and operational volume changes and ICoRV conditioning; however, mouth pressure during ICoTLC strongly correlated with EEVRCp (*rh*o = .613, p = .03) and EIVRCp (*rho* = .697, p = .01) changes. Considering the predicted value of maximal inspiratory pressure, the contraction intensity was approximately 30% and 60% of MIP and in the ICoTLC and ICoRV, respectively.

## Discussion

In the present study, the main findings were that only thixotropic conditionings performed from TLC increased EIV_CW_ and EEV_CW_ of PD patients, mainly due to changes in the upper ribcage compartments. Additionally, strong correlations were observed between the degree of inspiratory contraction and operational volume changes of the RCp compartment.

Disturbances of ventilation and breathing pattern are common in PD. They are related to the stiffness of the chest wall or factors affecting respiratory rhythm generation at the brain stem level [30, 31]. Difficulties in attaining maximum pressures and flows during rapid and forceful respiratory maneuvers may result from a complex restrictive-type defect [32, 33]. A link between respiratory muscle disturbance and impaired lung volumes has also been suggested [34]. In the present study, despite decreases in both FVC_%pred_ and FEV_1%pred_, the mean FEV_1_/FVC was higher than 70% of predicted in all patients, indicating a restrictive pattern [8, 22]. The mechanism of this pattern in PD patients is not well understood and has been attributed to respiratory muscle rigidity, bradykinesia, and loss of chest wall compliance [35]. MIP values were lower than MEP in 8 out of 12 patients, while SNIP values were consistently lower than the MIP, suggesting diaphragmatic impairment [5, 36].

Using OEP, Frazão et al. [7] observed that the upper ribcage compartments (RCp and RCa) of PD patients displaced less volume than healthy subjects, and the use of positive expiratory pressure led to increases in EEV_RCp_ and EEV_RCa_. Homma and Hagbarth [10] hypothesized that EEV of the upper ribcage compartments would also increase immediately after a thixotropic muscle conditioning with inspiratory muscle contraction at an inflated lung volume in healthy subjects. However, this was never tested in restrictive pulmonary diseases. Our group demonstrated results in a recent study conducted with healthy subjects, in which we observed a significant increase in EIV_CW_ compared with EEV_CW_ after performing three inspiratory contraction maneuvers from TLC and RV. The compartments that contributed most to EIV_CW_ results were the RCp and RCa. The significant increase of this variable in the RCa compartment suggests a possible thixotropic effect on the diaphragm. On the other hand, no differences were observed in EIV_CW_ and EEV_CW_ after expiratory contraction maneuvers starting from TLC or RV [15].

In our study, increases in EIV_CW_ and EEV_CW_ were observed mainly due to changes in volumes of the upper ribcage compartments, with no alterations in breathing patterns. Especially regarding EEV, the duration of EEV_RCp_ changes was longer than those observed for the EIV during DI_TLC_ and ICo_TLC_. In healthy, stiffness of the expiratory muscles is reduced following a large passive stretch of the muscles and voluntary lengthening contractions [10]; thus, favoring operational volume elevation. However, in PD patients, stretching a muscle may lead to increased muscle stiffness due to physical modifications of the muscles or neural mechanisms [37]. Several studies support the notion that non-neural alterations in the biomechanical properties of the stretched tissues may contribute to rigidity [38–41]. In this sense, we hypothesized that both muscle rigidity and impairment of expiratory muscle relaxation after large passive stretches have somehow accounted for the elevation of the EEV_RCp_ after the conditionings.

Interestingly, the increase in EEV_RCa_ and EIV_RCa_ suggests that the diaphragm may have also influenced the EEV_CW_ and EIV_CW_, respectively. Thus, increases in EEV_CW_ through diaphragmatic thixotropy may be a valuable therapeutic option in PD patients. Improvement in EEV and EIV strongly indicates improvement in tidal volume and ventilation efficiency. However, it is not known whether diaphragmatic thixotropy can act in patients with slight diaphragmatic impairment and those without diaphragmatic alterations [31, 42, 43].

Despite the lack of mouth pressure control, strong associations were observed between mouth pressure generation during ICo_TLC_ conditioning and EIV_RCp_ and EEV_RCp_. These results corroborate with Izumizaki et al. [44], suggesting that the strength of the respiratory muscles is an essential activator of thixotropy [45, 46] in PD patients. However, the after-effects observed during ICo_TLC_ did not differ significantly from those after the DI_TLC_ conditioning. Although plastic mechanical behavior of other respiratory tissues may account for the thixotropic inspiratory muscle behavior of the DI_TLC_ conditioning in healthy individuals [10, 47], in PD patients, this result is probably due to (or favored by) increased rigidity of central neural pathway origin [48, 49], enhanced passive stiffness [50], and the long-latency stretch response of muscles caused by atrophy and replacement by connective tissue [51–54]. Changes in intrinsic mechanical properties of skeletal muscles of PD patients lead to augmented stretch reflexes, which increase motor activity [49, 55–57] and may be potentiated by voluntary efforts [58]. Excessive motor unity recruitment and continuous electromyographic activity of rigid muscles are presented even at rest [51]. Although no reports are presented in literature about the increased passive stiffness of inspiratory muscles in PD patients, the scalene and parasternal muscles may show continuous abnormal activity due to extrapyramidal impairment [42]. Fontana et al. [59] observed that the electromyographic activity was slower in PD patients during forceful contractions suggesting impaired recruitment and derecruitment of abdominal motor units. Thus, if these responses are also valid for the inspiratory ribcage muscles, then the magnitude of the inspiratory effort may not be the only factor influencing the observed volume changes. Moreover, apart from the diaphragm that may not always be affected [42, 60], it cannot be maintained that ribcage respiratory muscles alone were responsible for the observed after-effects in PD patients. Activation of muscles other than those from the ribcage could have contributed to the results.

No changes in operational volumes were observed after thixotropic conditionings performed from RV. This result was expected since the main benefit of these conditionings would be the decrease of the EEV of patients with increased FRC [44]. Several studies reported the presence of an obstructive pattern in PD patients [61–64]; however, this was not the case for our patients. Although ribcage compliance was not measured, its decrease with a restrictive pattern and normal MEP may have probably accounted for the maintenance of operational volumes of RCp and RCa compartments after DI_RV_ and ICo_RV_ conditionings. Moreover, although changes in EEV_AB_ are insignificant and have no benefits for PD patients with a restrictive pattern, its decrease may be attributed to some thixotropic effect [44, 65]. Finally, the breath-holding during the conditionings may have affected breathing via central and peripheral chemoreceptors [31, 65]. Nevertheless, no significant changes were observed in breathing patterns and shortening velocity indexes after the conditionings, indicating no changes in the respiratory drive of PD patients.

From a clinical point of view, the results shown have attractive therapeutic potential. Thixotropic conditioning maneuvers can be used as a therapeutic strategy for patients with PD. They are easy to understand, low cost, easy to perform, and can be used in different environments, such as hospitals and at home. Clinical therapeutic strategies for patients with chronic diseases should involve treatments that are easy to incorporate into the daily lives of these patients, such as thixotropic conditioning maneuvers. From the point of view of clinical implications, new studies, especially clinical trials, should be carried out to confirm the results found.

## Study limitations

The main limitation of this study was the low number of patients included, Hoehn & Yahr scale class limited to II and III, and lack of a control group composed of healthy subjects. The lack of absolute volume quantification would also define the restrictive pattern completely. However, this is the first study that reports the after-effects of thixotropic conditionings in PD patients and using OEP. Regarding the protocol, more studies must be performed to understand whether the effects of the studied thixotropic conditionings are cumulative in PD patients and other restrictive diseases. Another limitation was the impossibility of confirming the presence or not of a restrictive ventilatory defect. Unfortunately, at the time of the study, TLC measurement was unavailable in our facility. Finally, another significant limitation was the lack of sEMG data acquisition. Data derived from sEMG could be relevant to monitoring muscle activation after a period of apnea.

## Conclusion

Thixotropic conditionings performed from an inflated chest wall position increase EEV_CW_ and EIV_CW_ of PD patients after the ten subsequent breaths, mainly due to changes in the upper ribcage volume.

## Supporting information

S1 Data (XLSX)
